# Retrograde elastic stable intramedullary nailing (ESIN) via a posterior approach to the radius as the principal cause of extensor pollicis longus tendon ruptures following pediatric forearm fractures

**DOI:** 10.1007/s00402-025-05950-5

**Published:** 2025-06-04

**Authors:** Marcel Winkelmann, Jonathan Biesemeier, Jorge Mayor Ramirez, Stephan Sehmisch, Jan-Dierk Clausen

**Affiliations:** Department of Trauma Surgery, Hannover Medical School, Carl-Neuberg-Straße 1, 30625 Hannover, Germany

**Keywords:** Radius fracture, ESIN, Elastic stable intramedullary nailing, EPL tendon rupture, Extensor pollicis longus tendon rupture

## Introduction

Forearm fractures are among the most common injuries in children, often resulting from falls or sports-related injuries. These fractures can vary in severity, ranging from hairline cracks to complete fractures, and may require different approaches to treatment. Surgical intervention is necessary for open fractures, irreducible fractures, and fractures with loss of reduction after closed treatment. Shaft fractures are usually treated with elastic stable intramedullary nailing (ESIN) that splints the fracture. Very distal fractures of the radius most commonly require percutaneous K-wire osteosynthesis. Metaphyseal fractures can be treated with ESIN or K-wires. However, ESIN gained popularity over the past decades [1]. Plate osteosynthesis may be reserved for complicated fractures and is comparatively rare these days. Depending on the preferred operation method the surgical approach is selected. Distal approaches comprise the radial and the posterior approach. The complication risk is generally low. However, there is a wide spectrum in the literature ranging from no complications up to 67% [2–5]. Typical approach related complications are superficial radial nerve palsy in the radial approach and extensor pollicis longus (EPL) tendon ruptures in the posterior approach [6]. In general, EPL ruptures are rare. However, incidence ranges from 0 to 16% in the literature [3, 5, 7] and the sample sizes are critically low. The total number of reported EPL ruptures in pediatric forearm fractures is lower than 100. This stands in marked contrast to clinical experience. Although extensor indicis proprius transfer provides satisfactory long-term extension of the thumb with reduced extension strength but negligible functional impact on the index [8, 9], EPL ruptures complicate postoperative recovery and affect outcome negatively.

Previous studies suggest an intra- or postoperative lesion of the extensor pollicis longus tendon most likely caused by implant material. The risk of EPL ruptures attributable to the posterior approach near Lister’s tubercle seems to be obvious. However, there are only single case reports about different approaches [10]. Furthermore, ESIN is more often accompanied by EPL rupture compared to K-wire osteosynthesis. But it is uncertain, whether this is due to a generally increased risk associated with ESIN or just due to frequency distribution, since ESIN is performed markedly more often than K-wire osteosynthesis. This leads to some major questions. Are EPL ruptures approach related? If so, are they procedure related and are there any references of intra- or postoperative tendon injury?

## Materials and methods

Therefore, we retrospectively analyzed postoperative course of children with forearm fractures treated at a single level 1 trauma center between 04/2011 and 09/2021. All patients had an outpatient postoperative follow-up and underwent implant removal at the indicated trauma center after bone healing.

Patients had to be under 16 years with radiologically open epiphyseal plates. Patients refused to participate or with incomplete data ware excluded.

Demographic and clinical data [age, gender, body mass index] was extracted from digital patient record. All fractures were diagnosed with conventional X-ray in two planes stored at the trauma center’s picture archiving and communication system. Fractures were classified by reference to AO pediatric comprehensive classification of long bone fractures (AO-PCCF) on a diagnostic monitor in adherence to DIN 6868 − 157. Soft tissue damage was graduated according to Gustilo-Anderson classification in case of open and to Tscherne-Oestern classification in case of closed fractures [11, 12]. Choices of implant and approach were assessed based on operative report and postoperative X-ray in two planes. EPL ruptures were detected by means of outpatient record and operative report regarding implant removal.

**Fig. 1:**
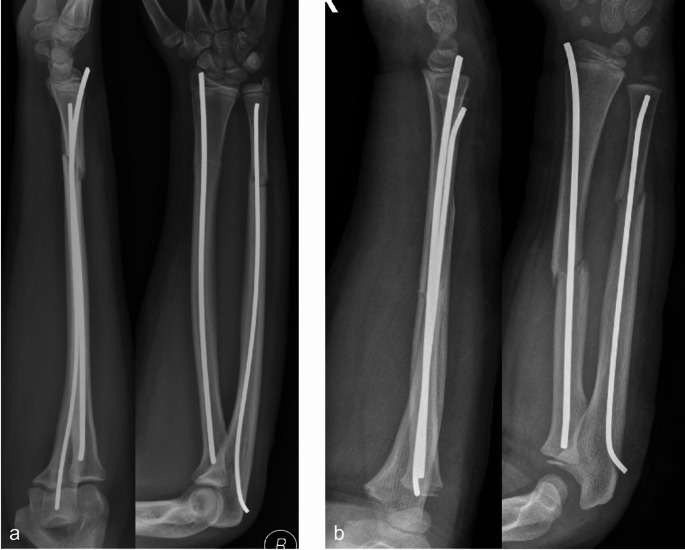
**a-b** Retrograde intramedullary nailing with posterior approach (**a**) and radial approach (**b**)

**Fig. 2:**
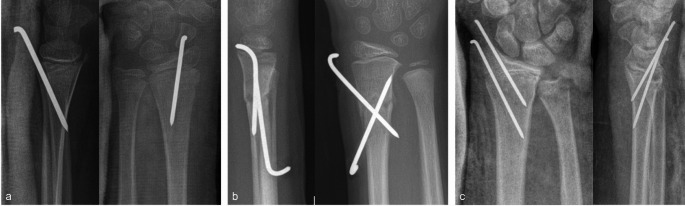
**a-c** Percutaneous K-wire osteosynthesis with posterior approach (**a**), radial approach (**b**) and trans-styloid approach (**c**)

Elastic stable intramedullary nailing was performed either via posterior (Fig. 1a) or radial approach at the distal radius (Fig. 1b). Percutaneous K-wire osteosynthesis was performed either in a Kapandji technique (Fig. 2a), in a X-shaped radial technique (Fig. 2b) or in a trans-styloid technique (Fig. 2c).

Statistical analyses were performed with IBM SPSS (Version 27, IBM Corp., Armonk, New York). All variables were checked for Gaussian distribution. Gaussian distributed variables were analyzed using parametric tests (Student’s t-test) and other variables were analyzed using non-parametric tests (Mann–Whitney U test for independent data; Wilcoxon test for dependent data). We used Fisher’s exact test (exact chi-squared test) in the analysis of contingency tables. Correlation analysis was made by means of Pearson correlation coefficient. Binomial logistic regression analysis was performed and odds ratios (OR) and 95% confidence intervals (CIs) were calculated. Significance was set at *p* = 0.05. The study has been approved by the institutional review board (Nr. 10153:BO_K2022) and patient’s parents had granted informed consent prior to inclusion.

## Results

**Fig. 3:**
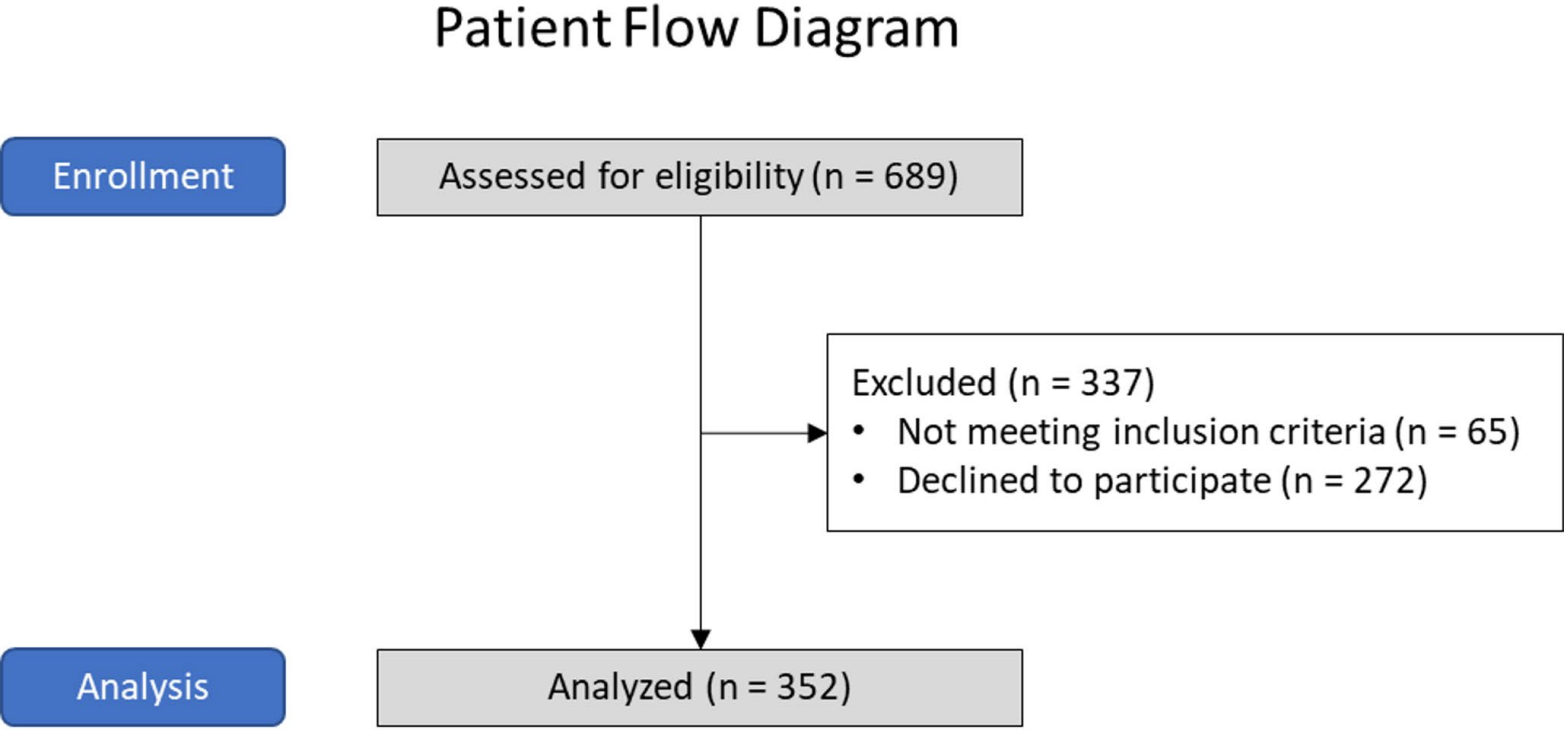
Patient flow diagram

352 participants were analyzed. Initially, 689 patients were assessed for eligibility. For more detailed information, please have a look at Fig. 3.

**Fig. 4:**
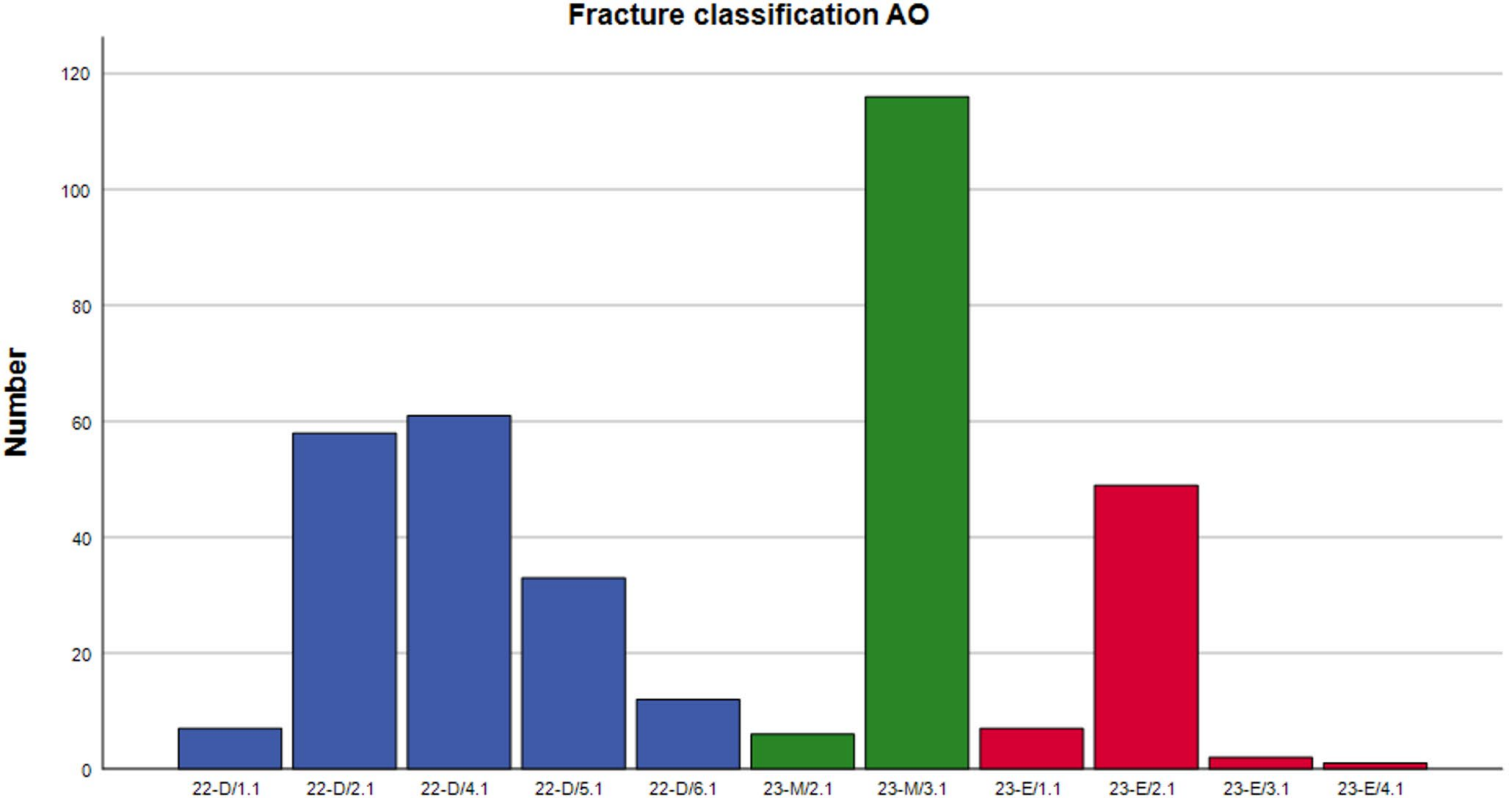
Fracture classification by reference to AO pediatric comprehensive classification of long bone fractures (AO-PCCF)

234 patients (66.5%) were male and 118 (33.5%) female with a mean age of 8.9 ± 3.7 years. Mean body mass index was 17.7 ± 7.6 kg/m². 198 patients (56.3%) sustained a distal forearm fracture and 154 (43.8) a forearm shaft fracture. Most common fractures were type AO M3.1 [*n* = 116 (33.0%)], D4.1 [*n* = 61 (17.3%)], D2.1 [*n* = 58 (16.5%)], as well as E2.1 [*n* = 49 (13.9)]. For detailed information, please have a look at Fig. 4. 159 fractures (45.2%) were treated with ESIN and 188 (53.4) with K-wires. 4 Patients were treated with a variable combination of ESIN and K-wires and 1 patient with ESIN and plate. 243 patients (69.0%) had a posterior and 169 patients (48.0%) a radial approach. Mean Follow-up was 3.7 ± 6.5 months.

9 patients (2.7%) sustained a postoperative lesion of the extensor pollicis longus tendon. 5 were complete and 4 were incomplete. All of them were treated operatively either with direct suture or extensor indicis proprius transfer.

There were no differences in age between patients with or without EPL lesion (9.5 ± 3.7 vs. 8.9 ± 3.7 years; *p* = 0.647) as well as in body mass index (16.6 ± 2.5 vs. 17.7 ± 7.7 kg/m²; *p* = 0.556). 6 of 9 patients with ruptured EPL tendon were females (*p* = 0.033). All EPL lesions occurred in patients with forearm shaft fracture (*p* < 0.001), treated with ESIN (*p* < 0.001) via a posterior approach (*p* = 0.042). EPL ruptures correlated with posterior approach (Pearson correlation coefficient 0.11, *p* = 0.042) and ESIN (Pearson correlation coefficient 0.18, *p* < 0.001). Female gender correlated with EPL ruptures as well (Pearson correlation coefficient 0.11, *p* < 0.033). However, it could not be proofed as an independent predictor in a logistic regression analysis (OR (95%-CI) = 3.0 (0.7–12.5), *p* = 0.135).

## Discussion

This study demonstrates an implant and approach relation of extensor pollicis longus tendon ruptures in pediatric patients. EPL ruptures are– essentially– referable to elastic stable intramedullary nailing via a posterior approach.

In the present study all EPL lesions occurred in patients with forearm shaft fracture. This appears curious at first sight. However, one must consider that shaft fractures were predominantly treated with ESIN and the typical approach at that trauma center during the study period was the posterior one. An additional impact of shaft fractures on EPL ruptures is not verifiable.

We did not find any directly fracture related injuries of the extensor pollicis longus tendon. The literature regarding spontaneous or trauma related EPL ruptures is scarce and comprises solely case reports [13, 14]. The exact mechanism of injury is not understood. Considering the frequency of pediatric forearm fractures and the functional impairment associated with EPL ruptures, one can assume that spontaneous trauma related ruptures are few and far between. Hence, there seem to be two major risk factors for EPL rupture. The posterior approach at the distal radius and the use of intramedullary nails. This is– generally– in accord with the literature. The vast majority of the published studies regarding pediatric EPL ruptures ascribed it to ESIN [2, 3, 15–18].

The posterior approach is more likely to cause problems with the extensor pollicis longus tendon [15]. This seems to be easily comprehensible, since the approach with the Lister’s tubercle as the major anatomic landmark is in direct proximity to the third extensor tendon compartment. Whether tendon rupture is due to intraoperative impairment due to manipulation or direct cut of the tendon or postoperative friction-related damage, respectively, remains debatable. Our own results suggest a postoperative chronic impairment. No rupture was present at the day of discharge and all ruptures have been diagnosed markedly later on. Basically, we cannot rule out an intraoperative incomplete damage, which is not obvious at the time of discharge. However, our data contradicts this assumption. One must consider that distal epi- and metaphyseal radius fractures have been largely treated with percutaneous K-wire osteosynthesis via posterior approach in a Kapandji technique at our trauma center over the study period. Therefore, there’s a large reference group in this study. With regard to comparable approaches, there were no EPL ruptures in the K-wire group. Intraoperative fracture manipulation should at least be comparable. Since ESIN was used in shaft fractures, manipulation certainly was more proximal and far away from typical region of tendon rupture at the extensor retinaculum. Hence, chronic friction-related impairment of the tendon seems to be quite likely. ESIN typically comprises a subcutaneous shortening of the intramedullary nail. K-wire osteosynthesis– at the study center– was solely performed percutaneous. This is in accordance with the literature. There are only very occasional reports about EPL ruptures in patients that were treated with K-wire [19–21]. In summary, EPL ruptures seem to be substantially due to chronic irritation caused by subcutaneous intramedullary nail near Lister’s tubercle (posterior approach). Consequently, the question that arises is, whether this is a necessary evil or an avoidable danger. In our opinion, it is largely avoidable. The treatment of shaft fractures with ESIN is generally accepted. To the best of our knowledge there are no studies that indicate a biomechanically inferiority of the radial approach to the distal radius in case of shaft fractures. Palsy of the superficial branch of the radial nerve is seldom and usually regresses spontaneously after implant removal. Therefore, the radial component of forearm shaft fractures can safely be treated with ESIN via a radial approach. But there are still the distal radius fractures. Epiphyseal fractures are commonly treated with percutaneous K-wires through the radial styloid process. This leaves the metaphyseal fractures of the radius. A retrograde intramedullary nail via a radial approach is biomechanically unstable. As an alternative to retrograde ESIN via a posterior approach one can antegrade splint the radius via a proximal or mid-shaft radial approach. From the clinical experience this procedure is steadily gaining popularity. The referred complication rate is low. However, literature is scarce and comparative analyses are lacking [22, 23]. Another option is a percutaneous K-wire osteosynthesis. This could be performed with intra-focal pinning as described by Kapandji or with extra-focal X-shaped pinning via a radial approach [24]. These techniques are reported to be reliable and show a lower complication rate compared to other techniques [25]. However, although there are a lot of studies about pediatric forearm fractures comparative analyses are lacking as well.

In general, most of the studies about pediatric EPL ruptures are longitudinal studies, this study as well. This makes it susceptible to a selection bias or loss to follow-up respectively. In our study roughly 40% of the patients refused to participate. Hence, we did not know whether there is a possible accumulation of EPL ruptures or complications. One cannot necessarily assume that complications are uniformly distributed. However, informed consent is gained prior to the osteosynthesis and we did not recognized cases of secondary withdrawal. For this reason, we are convinced that– considering the sample size– distribution of EPL ruptures is rather uniform. Another problem might be the diagnostic accuracy. A clinical examination of especially younger patients is challenging and makes it diagnostically less conclusive. Therefore, we cannot rule out undiagnosed ruptures. Due to the functional impairment of this rupture, it is rather unlikely from the clinical experience. Moreover, patients underwent outpatient examination by an experienced trauma surgeon with expertise in pediatric trauma. Since these fractures are treated only at specialized centers and the study center covers a wide catchment area and ensures the emergency treatment of pediatric trauma patients, the study population is quite representative. Age distribution is comparable to other studies [2, 3]. Gender distribution with male patients in the majority is in accordance with the literature and clinical experience. Therefore, we are convinced that our results are largely generalizable.

## Conclusion

In summary, retrograde intramedullary nailing with a subcutaneous shortened wire via a posterior approach seems to be the major risk factor of extensor pollicis longus tendon rupture after distal radius fracture. This complication seems to be evitable to a great extent. Shaft fractures can be treated with ESIN via a radial approach. Epiphyseal fractures can be treated with percutaneous K-wire osteosynthesis involving the radial styloid process. Only metaphyseal fractures could be a matter of debate. However, we are convinced that these fractures can adequately treated with antegrade intramedullary nailing or percutaneous K-wire osteosynthesis respectively. For this very reason, it is interesting how common retrograde intramedullary nailing via a posterior approach is. A possible explanation might be the sparse evidence base. Hence, we need comparative studies analyzing soft tissue complications as well as bony deformities and functional outcome of different osteosynthesis techniques especially in metaphyseal distal radius fractures.

## Data Availability

Data is avalaible with permission of the author at: https://doi.org/10.26068/mhhrpm/20250115-000.
